# Gan-Lu-Yin Inhibits Proliferation and Migration of Murine WEHI-3 Leukemia Cells and Tumor Growth in BALB/C Allograft Tumor Model

**DOI:** 10.1155/2013/684071

**Published:** 2013-03-17

**Authors:** Fon-Chang Liu, Chun-Hsu Pan, Ming-Tsung Lai, Shu-Jen Chang, Jing-Gung Chung, Chieh-Hsi Wu

**Affiliations:** ^1^School of Pharmacy, China Medical University, 91 Hsueh-Shih Road, Taichung 40402, Taiwan; ^2^Department of Pharmacy, Da Chien General Hospital, Miaoli 36052, Taiwan; ^3^Department of Pathology, Chung Shan Medical University, Taichung 40201, Taiwan; ^4^Department of Pathology, Chung Shan Medical University Hospital, Taichung 40201, Taiwan; ^5^Graduate Institute of Biological Science and Technology, China Medical University, Taichung 40402, Taiwan

## Abstract

The aim of this study was to explore the antitumor effect of Gan-Lu-Yin (GLY), a traditional Chinese herbal formula, on leukemia. Ethanolic extract of GLY was applied to evaluate its regulatory mechanisms in proliferation, migration, and differentiation of WEHI-3 leukemic cells as well as antitumor effect on BALB/c mice model. The results showed that GLY markedly reduced cell proliferation and migration with induced differentiation of WEHI-3 cells. The expression level of phosphorylated FAK, Akt, ERK1/2, and Rb was decreased p21 expression while level was increased in WEHI-3 treated with GLY. The results of cell cycle analysis revealed that GLY treatment could markedly induce G1 phase arrest and decrease cell population in S phase. Moreover, experimental results demonstrated that GLY decreased the protein expression and enzyme activity of MMP-2 and MMP-9. GLY treatment also reduced WEHI-3 leukemic infiltration in liver and spleen and tumor growth in animal model. Accordingly, GLY demonstrated an inhibitory effect on tumor growth with a regulatory mechanism partially through inhibiting FAK, Akt, and ERK expression in WEHI-3 cells. GLY may provide a promising antileukemic approach in the clinical application.

## 1. Introduction

In clinical, acute myeloid leukemia (AML), long-term survival is poor [1, 2]. Leukemia cancer is a highly complex, multitarget disease. Due to the defective cell signaling pathways, these cancer cells might alter their normal cycles of proliferation, transcription, growth, migration, differentiation, and death. The cells have the ability to avoid apoptosis and to proliferate in an uncontrolled manner leading to development of cancer. Arsenic trioxide (ATO) and all-*trans* retinoic acid (ATRA) have good clinical efficacy in treating the newly diagnosed and relapsed acute promyelocytic leukemia (APL, a subtype of AML) and AML [3]. Although Glasow et al. indicated that ATRA signaling plays a critical role in myelomonocytic differentiation [4], thus an important target for anti-AML therapy, several limitations constrained the use of ATRA in the treatment of AML. Long-term treatment of ATRA and ATO resulted in serious side effects including hypertension [5], interstitial pulmonary infiltrates, pleural and pericardial effusion, dyspnea, episodic hypotension, and acute renal failure [6]. Due to the drawbacks in treating AML, a new therapeutic agent with greater efficacy and fewer side effects for the treatment of this disease is urgently needed. 

Myeloblast cells (immature blood cells) possess two major functions to maintain normal physiological condition. Firstly, these cells are prone to cell proliferation to maintain the normal white blood cell number. Secondly, a cell can be differentiated to some mature cell with a specific function (e.g., white blood cell). Cell proliferation is usually counteracting cell differentiation. Therefore the balance between proliferation and differentiation of hematopoietic cell becomes very important [7]. It is generally believed that the proliferation, differentiation, and migration of leukemia cells were involved in the development of tumor growth and invasion. Therefore, modulation of leukemia cell growth, differentiation, and migration has important therapeutic implications. 

Many Chinese herbal medicines have been reported to have antileukemia cancer ability [8]. GLY consists of *Rehmannia glutinosa, Liriope spicata (Thunb.) Lour, Eriobotrya japonica (Thunb.) Lindl., Citrus sinensis Osbeck, Glycyrrhiza uralensis Fisch, Artemisia capillaris Thunb, Dendrobium nobile Lindl., *and* Scutellaria baicalensis Georgi.* In clinical practice, GLY has been routinely used in oral ulcer or swollen gums. This formula has been widely used to expel heat, to remove the dampness, to resolve inflammation, and to clean the blood according to the traditional Chinese medicinal prescriptions, Tai Ping Hui Min He Ji Ju Fang. Recently, we discovered that GLY has antiangiogenesis effect [9], and several other studies also showed that some single ingredient within GLY formula has biological effect on cell differentiation [10, 11]. To date, the effects of GLY on AML treatment are still unclear. Thereby, the present study was designed to evaluate the antitumor activities of GLY on BALB/c mice grafted with WEHI-3 cells and to investigate its inhibitory mechanisms *in vitro*. This study will help us to elucidate whether GLY treatment has potential to be an effective pharmacological reagent to treat leukemia cancer.

## 2. Materials and Methods

### 2.1. Materials

The ingredients of GLY were provided from Pharmacy Department of China Medical University Hospital, Taichung, Taiwan. Fetal bovine serum (FBS), penicillin G, and streptomycin were obtained from Invitrogen (Carlsbad, CA, USA). Primary antibodies against p-ERK1/2 (sc-7383) and p-Rb (sc-16670) were purchased from Santa Cruz Biotechnology (Santa Cruz, CA, USA). Antibodies against p-Akt (ab28821), p21 (ab379601), MMP-9 (ab58803), and *β*-actin (ab8226) were from Abcam (Cambridge, MA, USA). Phosphorylated-FAK (p-FAK, 8556) and MMP-2 (4022) were purchased from Cell Signaling (Beverly, MA, USA). Secondary antibodies conjugated with horseradish peroxidase were obtained from Santa Cruz Biotechnology. All other reagents were purchased from Sigma-Aldrich (Louis, MO, USA).

### 2.2. Preparation of GLY Extract

The ingredients of GLY formula were equally weighed (about 1 kg) and soaked in 10 liters of 50% ethanol solution (extractive solvent) for 3 days at room temperature. The solid residue of the above soaked herbs was filtered and discarded through a Buchner funnel lined with Whatman filter paper and the filtrate was concentrated to paste by vacuum distillation by using rotary evaporator (N-11, EYELA, Tokyo, Japan) and vacuum controller (VC-760, TAKARA, Tokyo, Japan) to maintain the desired pressure and temperature at 35°C with 40 mm Hg. The various concentrations of GLY were further diluted with Milli-Q water for the further use.

### 2.3. Cell Culture

WEHI-3 cells were purchased from Food Industry Research and Development Institute (Hsinchu, Taiwan). The cells were seeded on 75 cm^2^ tissue culture flasks and culture condition as described previously [12]. 

### 2.4. Cell Viability Assay

Cell viability was determined by the previous study with minor modification [13]. Cells were seeded on 6-well plates (2 × 10^5^ cells/well) and exposed to various concentration of GLY extract for 24 h in a total volume of 1 mL culture medium with 10% FBS supplement. After 24 h treatment, an aliquot of cells suspension was mixed with 0.4% trypan blue (GIBCO, USA) and to count cells with a hemocytometer while keeping a separate count of the blue cells. The cells that failed to exclude the dye were considered nonviable; therefore, the data is expressed as a percentage of viable cells.

### 2.5. Cell Cycle Analysis

Cells were seeded on 6-well plates (2 × 10^5^ cells/well) and exposed to various concentrations of GLY extract for 24 h in a total volume of 1 mL culture medium with 10% FBS supplement. After 24 h treatment with GLY, WHE-3 cells were harvested and washed with cold 1× PBS and then fixed with 70% ice-cold ethanol overnight. After ethanol was removed by centrifugation, pellets were resuspended in 500 *μ*L of DNA staining buffer (4 *μ*g/mL of propidium iodide, 1% (v/v) Triton X-100, and 0.1 mg/mL of RNase A) and then incubated for 30 min at room temperature in the dark and followed by flow cytometry using FACSCanto (BD Biosciences, San Jose, CA, USA). Cell cycle profile was analyzed using ModFit LT Program (Verify Software House, Topsham, ME, USA).

### 2.6. Cell Differentiation Assay

Differentiation of WEHI-3 cells was assessed using the NBT (nitroblue tetrazolium) reduction test. The assay was carried out according to the method with a minor modification [14]. WEHI-3 cells were seeded on a 24-well plate (2 × 10^5^ cells/well) and exposed to GLY for 48 h and then cells were harvested by centrifugation. After that, the experiment was performed by adding 200 *μ*L PBS containing 1 mg/mL NBT and 2 *μ*g/mL phorbol myristate acetate solution at 37°C for 1 h in darkness. The mix solution was transferred to 96-well plate then the cells were incubated for 1 h at 37°C in 5% CO_2_. Following incubation, the medium was aspirated from the wells, cells washed twice with warm PBS, and then intracellular formazon crystals of pellets were dissolved by the addition of 100 *μ*L of DMSO to each well. The absorbance read at 570 nm on a spectrophotometer. The NBT-positive cells containing purple formazan deposits were identified using a light microscopy at 400x magnification.

### 2.7. Western Blot Analysis

 The tumor tissues (50 mg) were homogenized in 250 *μ*L lysis buffer (iNtRON, Gyeonggi-do, Republic of Korea), and WEHI-3 cells (1.6 × 10^6^ in 10 cm culture dish) were lysed with 0.07 mL lysis buffer. Tissue or cell lysate was incubated on ice for 30 min and insoluble components were removed by centrifugation at 13,000 rpm for 10 min at 4°C. The supernatants were collected and their protein concentrations were determined by using the Bradford protein assay kit (Bio-Rad Laboratories, USA). Total proteins (50 *μ*g) were resolved in sodium dodecyl sulfate-polyacrylamide gel electrophoresis (SDS-PAGE) followed by blotting to PVDF (polyvinylidene fluoride) membrane (NEF1002001PK; Perkin Elmer, Boston, MA, USA). After total protein being successfully transferred on PVDF membrane, nonspecific binding sites were blocked by incubating membranes in 5% (w/v) of nonfat milk dissolved in 1 × PBS with 0.1% of Tween 20 (PBST buffer). Primary antibodies against p-FAK, p-Akt, p-ERK1/2, p-Rb, p21, MMP-2, and MMP-9 reconstituted in PBST buffer were added and incubated overnight at 4°C. After being washed with PBST buffer, respective protein bands were visualized by using ECL reaction (Amersham, Arlington Height, IL, USA). The luminescence signal was acquired and analyzed by Fujifilm LAS-4000 system (San Leandro, CA, USA). The protein level was normalized to *β*-actin and presented as fold change to control group.

### 2.8. Gelatin Zymography

The test was performed as our previous study with minor modification [15]. WEHI-3 cells were seeded (6 × 10^5^ cells/well) and incubated with GLY (0, 0.25, 0.5, and 1 mg/mL) for 24 h under serum-free medium. The supernatant was collected by centrifugation at 10,000 rpm for 5 min. Thirty-two microliters of samples were mixed with eight microliters of 5X nonreducing loading buffer (12.5% bromophenol blue, 10% SDS, 0.5 M Tris-HCL pH = 6.8, 50% glycerol) and then electrophoresed in 8% polyacrylamide gel with 0.1% (w/v) gelatin. After that, the gel was washed twice (30 min/time) by 2.5% Triton X-100, incubated with zymography reaction buffer (1.54 mM NaN_3_, 12.6 mM CaCl_2_, 40 mM Tris-HCl, pH 8.0) for an additional 18 h at 37°C, and stained with coomassie blue R-250 (0.125% coomassie blue R-250, 50% methanol, 10% acetic acid) and destained with destaining solution (methanol : acetic acid :  water = 40 : 10 : 50, v/v). Densitometric analysis of the gelatinase-digested (clear) bands was quantified by using ImageJ software (NIH, Bethesda, MD, USA).

### 2.9. Cell Migration Assay

The examination was tested as the method reported by Li et al. with minor modification [16]. The transwell cell culture chambers (5 *μ*m pore size, Corning Costar, Cambridge, MA) were applied to measure the migration effect of WEHI-3 cells treated with GLY. WEHI-3 cells resuspended in serum-free culture medium and placed in the upper chamber of the transwell insert (1 × 10^6^ cells/well) and incubated with GLY (0, 0.25, 0.5, and 1 mg/mL) and culture medium (with 10% FBS as a chemoattractant) were added to the lower chamber. The plates were incubated in a humidified atmosphere with 95% air and 5% CO_2_ at 37°C for 4 h. The nonmigrated cells on the inner surface of the upper chamber were removed by wiping with a cotton swab, and migration cells on the outer surface of the upper chamber were fixed with 75% ethanol solution and stained with Giemsa solution. The migrated cells were determined using a light microscope (40x) and NIH Image software (Image J).

### 2.10. HPLC-ESI/MS Analysis of GLY Extract

The examination was performed according to our previous study with minor modification [9]. The external standards and GLY extract were prepared as concentration of 100 *μ*g/mL and 2 mg/mL in HPLC grade methanol, respectively. Before HPLC analysis, sample solutions were filtered through a 0.2 *μ*m filter. Total volume of 10 *μ*L sample was loaded into HPLC column to measure the relative content of naringin, naringenin, and vanillin within GLY extract according to the concentration of external standards. Separations were accomplished on LiChroCART 250-4 C18 HPLC-cartridge (5 *μ*m; Merck, Whitehouse Station, NJ, USA). The separation conditions of HPLC analysis for examined compounds were described in Table 1. According to the HPLC separation condition of standard compounds, three candidate fractions (collection period: retention time ±5 min) were harvested by HPLC system. These collected fractions were concentrated by evaporation and redissolved in methanol and then further examined by electrospray ionization-ion trap mass spectrometry (ESI-ion trap MS) system (HCT ultra-PTM Discovery system; Bruker Daltonics, Billerica, MA, USA) to identify the marked (or potential) compounds within GLY extract. Capillary voltage was 4000 V, capillary exit offset 220 V, skimmer potential 40 V, and the trap drive value was 78. Conventional ESI-MS/MS data were recorded using a scan range of 100–1000 m/z. Nebulizer (nitrogen) pressure was 10 psi, dry gas (nitrogen) flow 5 L/min, and dry temperature 300°C.

### 2.11. WEHI-3 Allograft Tumor Model of BALB/c Mouse

The animal experiments were approved by the Institutional Animal Care and Use Committee of China Medical University (approval ID: 101-238-C). All animal care followed the institutional animal ethical guidelines of China Medical University. Twenty eight BALB/c male mice (average 25–28 g; 8 weeks old) were obtained from Laboratory Animal Center, College of Medicine, National Taiwan University (Taipei, Taiwan). The mice were randomly divided into 4 groups (*n* = 7/group) and were kept on 12 h light/dark cycle at 25°C. Group *Ι* (normal control group) was only given distilled deionized water (DDW). Group *Ι*I (untreated group) was subcutaneously grafted with WEHI-3 cells (2 × 10^6^ cells in 100 *μ*L). Group III was grafted with WEHI-3 cells and then treated with GLY (0.75 g/kg). Group IV was grafted with WEHI-3 cells and then treated with GLY (1.5 g/kg). GLY was diluted in DDW and administrated by oral gavages. The mice were treated daily for 2 weeks and then sacrificed. The livers, spleens, and tumors were obtained and weighed individually.

### 2.12. Histopathology Examination

The examinations were carried out according to our previous study with minor modification [17]. Tissue samples were fixed in 4% formaldehyde and embedded in paraffin. Each tissue sample was cut into 5 *μ*m section and stained with hematoxylin-eosin (H&E) method. The histological images were photographed under a light microscopy at 400x magnification. All tissues for histopathological examination and identification of the leukemia cancer cells in the tissue section are looked at under a microscope by a pathologist, a doctor who has special training in laboratory diagnosis of cancers.

### 2.13. Measurement of Liver Index and Spleen Index

The following formulae can be used for the calculation of spleen index and liver index, spleen index = spleen weight (g)/body weight (g) × 100; liver index = liver weight (g)/body weight (g) × 100.

### 2.14. Statistical Analysis

 All data are presented as mean ± SD for triplicate experiments. Statistical significance was evaluated by one-way ANOVA. A value of *P* < 0.05 was regarded as being statistically significant. 

## 3. Results

### 3.1. Inhibitory Effect of GLY on Cell Proliferation of WEHI-3

**Figure 1:**
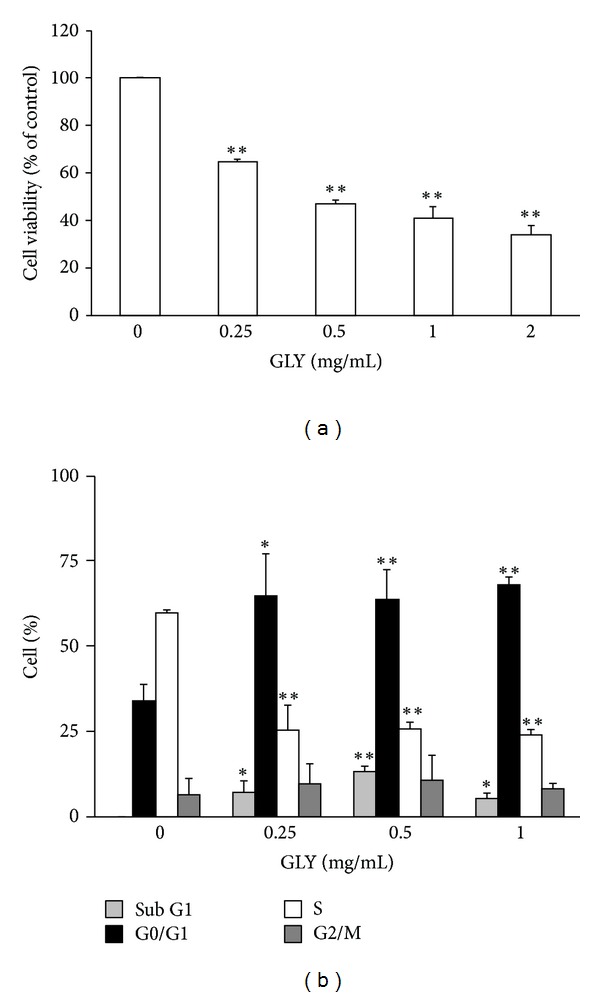
GLY affects cell viability and cell cycle distribution in WEHI-3 cells. WEHI-3 cells were incubated with various concentrations (0.25, 0.5, 1.0, and 2.0 mg/mL) of GLY containing 10% FBS for 24 h treatment. The percentage of cell viability was calculated according to the values of control group (10% FBS-treated group) as 100% (a). The distribution of cells undergoing various phases of the cell cycle was determined in WEHI-3 cells treated with 0.25, 0.5, and 1.0 mg/mL GLY for 24 h (b). The percentage of celld of each treatment was presented as the histogram. Histograms of all values are expressed as the mean ± SD. *P < 0.05 and **P < 0.01 compared with the control group.

The antiproliferative effect of GLY was analyzed on WEHI-3 treated with 10% FBS to stimulate cell growth. After 24 h treatment with GLY, the results showed that the growth effect of WEHI-3 proliferation could be markedly attenuated by GLY (Figure 1(a)) in a dose-dependent manner as compared with control group (0 mg/mL, *P* < 0.05; *n* = 3). 

### 3.2. Effect of GLY Extract on Cell Cycle Distribution of WEHI-3

The cell cycle distribution of WEHI-3 was examined by flow cytometry on cells treated with various concentrations (0.25, 0.5, and 1.0 mg/mL) of GLY for 24 h. Our result indicated that 24 h treatment of GLY could markedly arrest cell cycle distribution at G0/G1 phase and decrease at S phase (Figure 1(b), *P* < 0.05; *n* = 3) as compared to those of control group (0 mg/mL, PBS treated only). The cell population at G0/G1 phase was increased from 33 to 67% by GLY treatment. 

### 3.3. GLY Induces Monocytic Differentiation in WEHI-3 Cells

**Figure 2:**
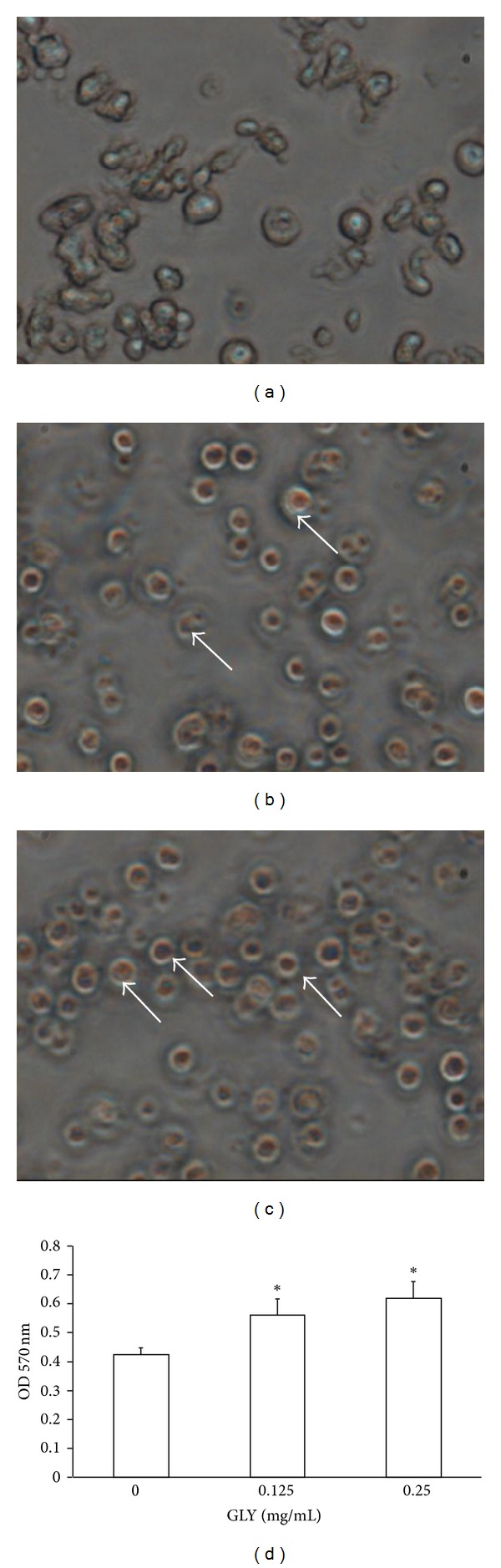
GLY induces differentiation in WEHI-3 cells. WEHI-3 cells were treated with 0, 0.125, and 0.25 mg/mL GLY for 48 h and then examined by NBT reduction assay showed insoluble and visible purple formazan precipitates in GLY-treated WEHI-3 cells ((a) 0 mg/mL GLY; (b) 0.125 mg/mL GLY; (c) 0.25 mg/mL GLY). The pictures were taken under a microscope at 400x magnification. The OD. 570 nm of each treatment was presented as the histogram. All values are expressed as the mean ± SD. * indicates *P* < 0.05 as compared with control group (0 mg/mL) (→: NBT-positive cells).

NBT-reducing activity, the typical markers of myelomonocytic differentiation, was examined. Our data showed that 48 h of GLY treatment induced NBT positive cell expression as demonstrated by the presence of intracellular purple formazan deposit (Figure 2). WEHI-3 cells were differentiated by GLY in a dose-dependent manner. Incubation of the cells with 0.125–0.25 mg/mL of GLY for 48 h increased the cell differentiation by 32–45% compared to those of control group (0 mg/mL, PBS treated only), respectively. 

### 3.4. Inhibitory Effect of GLY Extract on Cell Migration of WEHI-3

**Figure 3:**
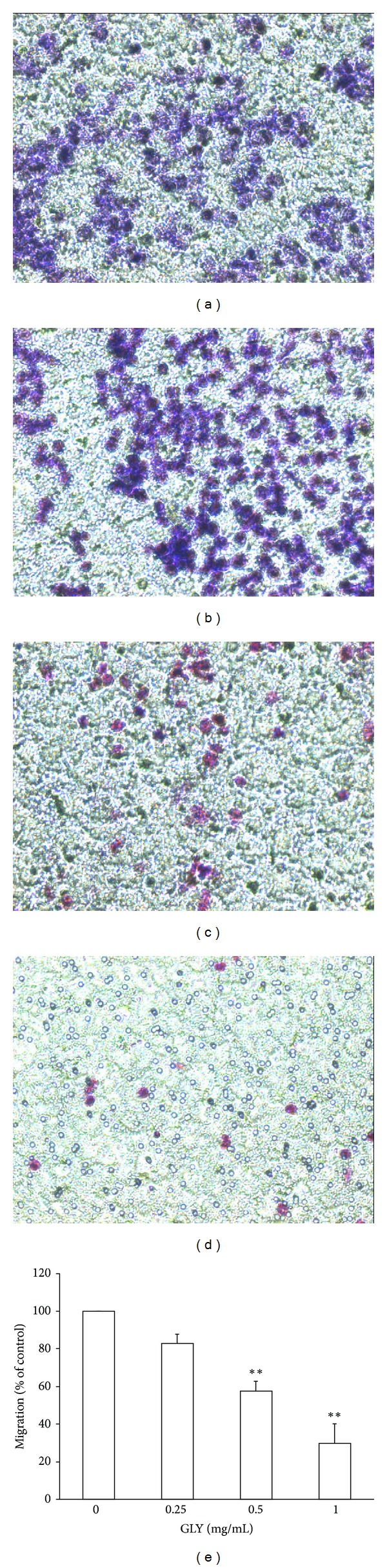
Effect of GLY on WEHI-3 migration was measured by transwell assay. Cells were incubated with various concentrations (0, 0.25, 0.5, and 1.0 mg/mL) of GLY for 4 h and were measured by the transwell assay as described in Materials and Methods ((a) 0 mg/mL group, (b) 0.25 mg/mL group, (c) 0.5 mg/mL group, and (d) 1 mg/mL group). The pictures were taken under a microscope at 200x magnification, and the migrated cells were calculated. * indicates *P* < 0.05 as compared with control group (0 mg/mL). Data are presented as the mean ± SD of three separate experiments (e).

Migration assays were performed using transwell assay. The antimigratory effect of GLY was analyzed on WEHI-3 cells treated with 10% FBS to stimulate cell migration. After 4 h treatment of GLY, 10% FBS stimulation could largely increase cell migration of WEHI-3, and this migratory effect could be markedly attenuated by GLY treatment (Figures 3(b), 3(c), and 3(d)). In the present study, GLY was found to inhibit WEHI-3 migration in a dose-dependent manner as compared with control group (Figure 3(e), *P* < 0.05; *n* = 3).

### 3.5. GLY Decreased Gelatinase Activity in WEHI-3 Cells

**Figure 4:**
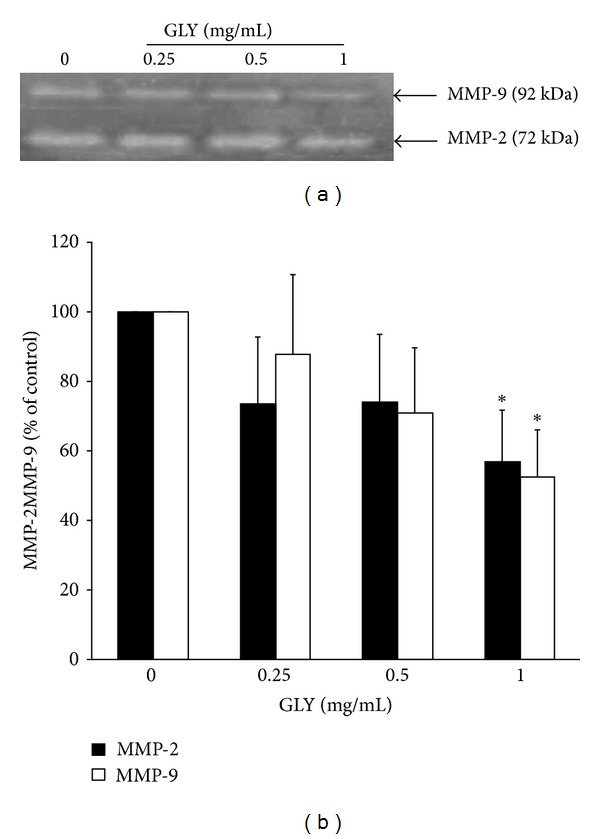
Effects of GLY on MMP-2 and MMP-9 activities of WEHI-3 cells. Cells were treated with various concentrations (0.25, 0.5, and 1.0 mg/mL) of GLY for 24 h. MMP-2 and MMP-9 activities were determined by gelatin zymography. The activities of these proteins were subsequently quantified by densitometric analysis. Values (mean ± SD, *n* = 3) differ significantly (*P* < 0.05) (lower case for MMP-2 and upper case for MMP-9).

To investigate the effect of GLY on gelatinase activity, the WEHI-3 cells were treated with 0.25, 0.5, and 1.0 mg/mL of GLY for 24 h, and the accumulated amount of active MMP-2 and MMP-9 protein in cultured medium of WEHI-3 cells was examined (Figure 4). The result indicated that 1.0 mg/mL of GLY could markedly decrease the activity expression of MMP-2 and MMP-9 in culture medium as compared with control group (Figure 4, *n* = 3).

### 3.6. GLY Inhibited the Expression of Migration- and Cell Cycle-Associated Proteins in Allograft Tumor Samples

We analyzed the protein expression levels of gelatinases and p21 proteins of tumor samples in allograft tumor mice model after 14 days of oral administration of GLY (0.75 and 1.5 g/kg). Experimental results showed that 1.5 g/kg of GLY could significantly reduce the protein levels of MMP-2 and MMP-9 but increase p21 expression as compared with control group (see Supplemental S1 in Supplementary material available online at http://dx.doi.org/10.1155/2013/684071, *n* = 3). 

### 3.7. Regulation of GLY on Proliferation, Migration and Cell Cycle-Associated Proteins in WEHI-3 Cells

**Figure 5:**
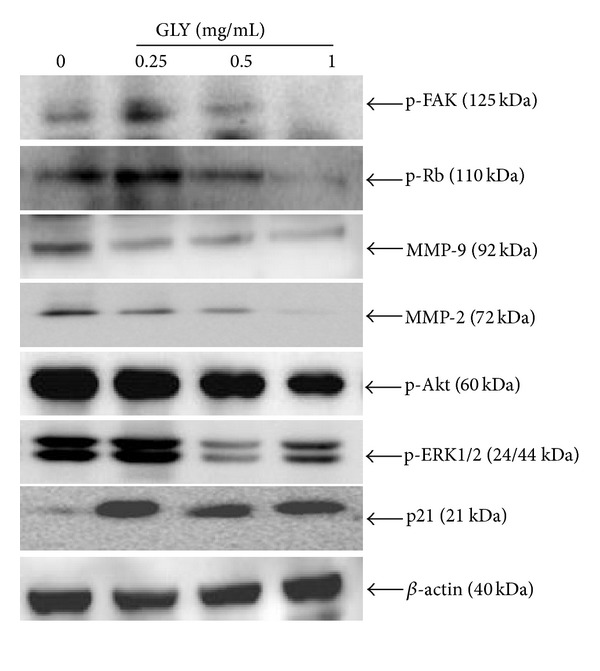
Effects of GLY on the phosphorylation of FAK and its downstream targets Akt, ERK1/2, gelatinase of MMP-2, MMP-9, and cell-cycle-related protein Rb and p21 in WEHI-3 cells. WEHI-3 cells were incubated with several concentrations of GLY for 24 h to examine the protein expression level of p-Rb, p-FAK, MMP-2, MMP-9, p-Akt, p-ERK1/2, and p21 proteins. Each value represents the average of three independent experiments in Table 4.

The WEHI-3 cells were treated with 0.25, 0.5, and 1.0 mg/mL of GLY for 24 h. The results indicated that GLY could markedly decrease p-FAK, p-Akt, p-ERK1/2,MMP-2, MMP-9, and p-Rb expression levels but increase p21 protein expression as compared with control group (Figure 5 and Table 4, *n* = 3).

### 3.8. Antitumor Effect of GLY in Murine WEHI-3 Leukemia Model

The result showed that 2-week treatment of GLY (0.75 g/kg) significantly reduced tumor size by 61% as compared to untreated group (Table 2, *P* < 0.05). Furthermore, experimental results also showed that GLY (1.5 g/kg) was effective at decreasing the leukemic infiltration-induced swelling of liver and spleen by 18.6% and 21.4%, respectively, as compared to untreated group (*P* < 0.001).

### 3.9. Effects of GLY on Leukemic Infiltration-Induced Swelling of Liver and Spleen in Murine WEHI-3 Leukemia Model

**Figure 6:**
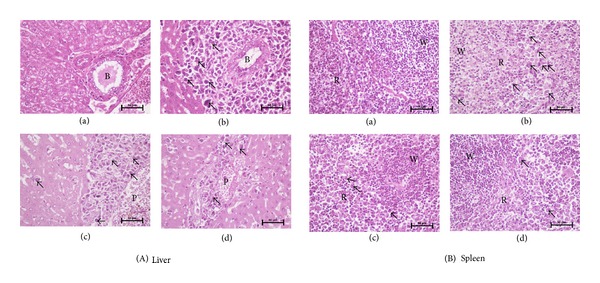
Histopathological examinations of liver and spleen tissues in WEHI-3 allograft tumor model. Tissue sections were stained with hematoxylin-eosin to evaluate the leukemic infiltration. The pictures ((a) normal control group; (b) untreated group; (c) 0.75 g/kg GLY group; (d) 1.5 g/kg GLY group) were taken under a microscope at 400x magnifications. Arrowheads indicate the infiltrated WEHI-3 leukemic cells within the liver and spleen tissues. W: white pulp of spleen; B: bile duct of liver; R: red pulp of spleen; P: portal vein of liver.

Figure 6(A)-(b) showed that infiltrated WEHI-3 cells were evident in the portal area of liver, resulting in surrounding enlargement of the bile duct and portal vein. In contrast, Figure 6(A)-(d) displayed that 1.5 g/kg GLY can improve the phenomenon induced by leukemia infiltration in the portal area of liver. Similarity, the results also showed that leukemia infiltration in the red pulp of spleen was also obviously reduced by GLY treatment (Figure 6(B)-(c) and 6(B)-(d)).

### 3.10. Influence on the Weight of Immune Organs of Mice

The GLY group showed a decrease in the weight of the spleen and liver in tumor-bearing mice (Table 2, *P* < 0.05), while the untreated group had a significantly higher liver weight and spleen weight (Table 2, *P* < 0.05).  Table 3 shows the values of spleen index and liver index for all experiment groups after 2 weeks of treatment. The spleen index and liver index in GLY (1.5 g/kg) were significantly decreased as compared to untreated group (0.58 ± 0.12 versus 0.44 ± 0.07; 7.04 ± 0.24 versus 5.86 ± 0.15). 

### 3.11. Qualification of the Marked Compounds within GLY by Liquid Chromatography/Mass Spectrometry Analysis (LC/MS)

**Figure 7:**
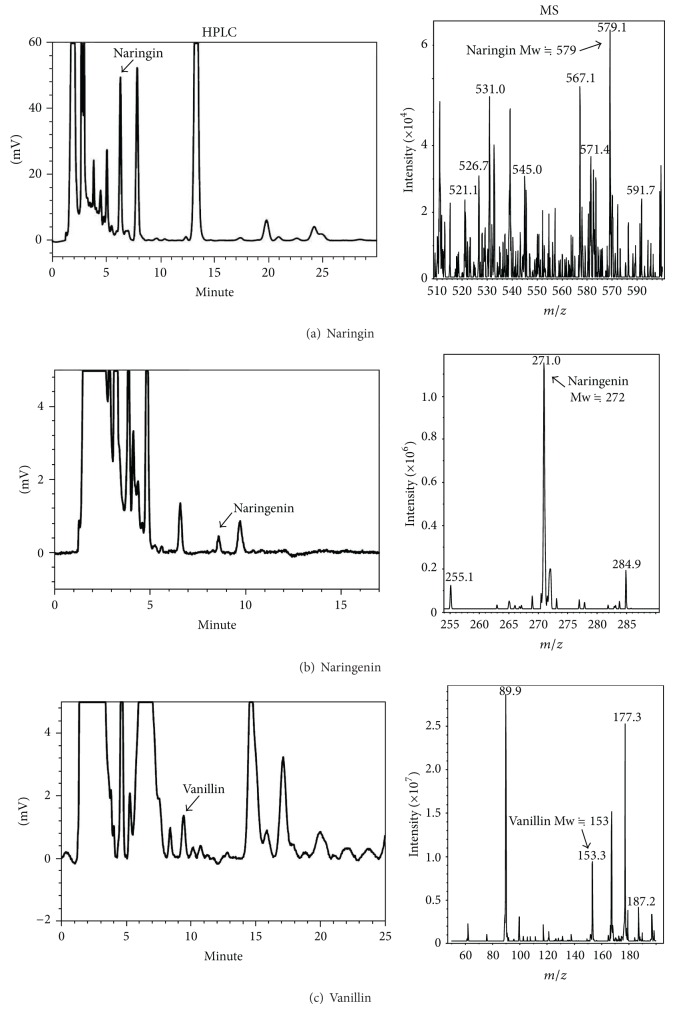
Representative HPLC and MS chromatograms of the marked compounds within GLY. The separation conditions were described in the Materials and Methods. The relative content of naringin, naringenin, and vanillin within GLY was measured by HPLC. Three HPLC fractions of GLY were collected according to the retention time of standard compounds to further examine by ESI-MS to confirm molecular size of the suspected compounds, naringin, naringenin, and vanillin.

Some of the index compounds (e.g., naringin, naringenin, and vanillin) from single ingredient within GLY formula were identified by LC/MS to be the index compounds for quality confirmation of extraction procedure for each batch (Figure 7). Our result found that naringin, naringenin, and vanillin could be detected in GLY extract, and the content of naringin, naringenin, and vanillin was calculated to be 13, 0.32, and 0.35 mg/g of GLY extract, respectively (Table 1).

## 4. Discussion

Recently, Recher et al. mentioned that enhanced expression of FAK in AML promoted cell migration with poor prognosis [18]. Upregulation of FAK may activate antioxidant enzymes and suppress lipid peroxidation, resulting in antiapoptosis against oxidative stress in human HL-60 cell line [19]. Furthermore, FAK also induced podosome rosettes assembly leading to cell motility and extracellular matrix degradation [20]. On the other hand, the FAK downstream regulator, Akt, also played a critical role in intracellular activation of cell growth and antiapoptosis for cancer survival via mTOR and NRF2 signaling pathway. FAK/PI3 K/Akt was also found to be involved in the regulation of MMP-2 and MMP-9 activities in different cell types [15, 21–23]. In the present study, we found GLY extract not only decreased p-FAK but also reduced p-Akt protein expression in the cell lysate (Figure 5). This effect might explain why GLY extract could significantly affect several biological functions of leukemia cells, including cell proliferation and migration. 

 Extracellular signal-regulated kinase (ERK) belongs to the family of mitogen-activated protein kinases, which play a critical role in the induction of intracellular activation including proliferation and metastasis in different cancer cells [24, 25]. In addition, ERK can be constitutively activated in leukemic progenitor cells [26], and inhibition of MEK/ERK signaling leading to apoptosis of AML cells may be a therapeutic remedy in the treatment of leukemia. Moreover, downmodulation of ERK protein kinase activity was found to inhibit VEGF secretion by human myeloma cells as well as myeloma-induced angiogenesis [27, 28]. Furthermore, disruption of retinoblastoma-lamin A complexes and enhanced Rb phosphorylation by ERK can cause E2F release from Rb-E2F complex and thus promote cell cycle entry and cellular transformation resulting in tumor cell proliferation [29]. Beside, down-regulation of MMP-2 and MMP-9 expression with subsequent decrease in leukemia cancer invasion by caffeine has been attributed to Ca^2+^/ROS-mediated suppression of ERK signaling pathway [30]. In the present study, we found that GLY could reduce protein level of p-ERK1/2 and p-Rb expression in cell lysate (Figure 5), suggesting the important role of GLY in cancer progression.

Furthermore, cell cycle advancement also played a critical regulatory mechanism in cell growth. The cell cycle was regulated via Cdk inhibitors to control cyclin-dependent kinases (Cdks) expression. p21, one of the Cdk inhibitors, was found to regulate the cell cycle progression via interaction with a cyclin-cdk complex to block the kinase activity of Cdk. Activation of p21 by vitamin D3 has been reported to induce myelomonocytic cells differentiation [31]. Cyclin-cdk complexes including cdk4 and cdk2 complexes are activated early in the G1 phase. Overexpression of p21 has been known to block the progression of cells from G1 to S phase by counteracting the cdk4 and cdk2 complexes. Furthermore, several studies indicated that Akt could stabilize p21 protein via directly phosphorylating p21, and then increased Cdk activity has been found to stimulate DNA synthesis and cellular proliferation [32, 33]. p21 can also elicit dephosphorylation of Rb and inactivates Rb by degradation in human fibrosarcoma cells [34]. In contrast to phorbol 12-myristate 13-acetate-(PMA-) induced leukemia differentiation process, PI3K was found to suppress PMA-induced p21 expression and to reduce cell differentiation of leukemia cells [35]. In the present study, we found that GLY not only increased p21 protein expression in tumor lysate (Supplemental S1) but also increased p21 as well as decreased p-Akt expression in cell lysate (Figure 5).

Leukemia differentiation has also been regarded as an alternative approach for leukemia treatment. ROS (reactive oxygen species) may play an important role in the redifferentiation induced by drugs such as arsenic trioxide. Actually, a relatively high level of ROS is necessary for cell differentiation and animal development [36]. Recently, a study indicated ROS such as H_2_O_2_ can activate NADPH oxidase, leading to O_2_^∙^^−^ product, followed by ERK activation and ultimately resulting in the differentiation of HL-60 cells [37]. In this study ROS production was detected by nitroblue tetrazolium (NBT) assay. According to the presence of intracellular purple formazan deposit (Figure 2), our data showed that GLY markedly increase ROS production after 48 h of treatment. This indicated that GLY-induced differentiation was dependent on ROS production. This further supports previous data obtained in the K562 and HL-60 cell lines with various inducers, including anthracyclines, butyric acid, Ara-C, ADP-Fe^2+^, H_2_O_2_, and aclarubicin [38]. In the present case of GLY-induced differentiation, ROS generation will probably stimulate specific gene expression through redox sensitive transcription factors (NF-*κ*B, AP-1, NF-E2, etc.). The involvement of these factors has been demonstrated in the erythroid differentiation of K562 cells. Moreover, other bioactive compounds of GLY, such as baicalin and chlorogenic acid, have been reported to increase the ROS generation and then affect different type leukemia cell growth [39, 40]. The above studies might partially support that GLY could significantly affect WEHI-3 cells on differentiation. As shown in Figure 1(b), WEHI-3 cell population at G0/G1 phase was increased from 33 to 67% by GLY treatment. These data showed that GLY was able to arrest cell cycle of leukemia cells with subsequent differentiation to macrophage-like cells.

Moreover, MMPs of leukemia cancer cells are the important regulatory factors for cell migration, invasion, and angiogenesis [41, 42]. Feng et al. found that MMP-2 and -9 can increase the permeability of blood-brain barrier by disrupting the tight junction proteins resulting in leukemic cells infiltration to CNS [43]. Therefore, drugs capable of inhibiting leukemia cancer invasion, infiltration, and migration are effective in protecting cancer progression. In the present study, we found that GLY not only attenuated enzyme activities and protein expression level of MMP-2 and MMP-9 in cell lysate, respectively (Figures 4 and 5), but also reduced MMP-2 and MMP-9 protein expression in tumor lysate (Supplemental S1). This finding provided one of the molecular mechanisms why GLY could markedly affect several biological functions of leukemia cells including migration and invasion.

Recently, Lin et al. indicated that GLY was composed of 14 main components via LC/MS and inductively coupled plasma MS (LCP/MS) analysis, including baicalin, baicalein, oroxylin A-7-O-glucuronide, wogonin-7-O-glucuronide, wogonin, and oroxylin A liquiritigenin, liquiritin, glycyrrhizic acid [44]. Our previous study has found baicalein, chlorogenic acid, and glycyrrhizic acid as extra components of GLY by LC/MS analysis [9].

Liu et al. reported that baicalin and baicalein, two flavonoids compounds, have inhibitory effects on endothelial cell proliferation, migration, and differentiation [45]. Moreover, it was found that CD11b was highly expressed on myeloid cells when they were differentiated toward either macrophages or granulocytes. Ikezoe et al. indicated that baicalin can induced differentiation of HL-60 cells with overexpression of CD11b [46] and wogonin upregulates phospholipid scramblase 1 gene expression and then induces the granulocytic differentiation of NB4 promyelocytic leukemia cells [47]. Although chlorogenic acid has been known as a caffeic acid-derivative compound, there were no direct lines of evidence to point out its antiangiogenic effect. However, the previous literature indicated that it is a new type and strong matrix metalloproteinase-9 inhibitor which might prevent the invasion and metastasis of malignant cancer cells [48, 49] and chlorogenic acid has inhibitory effects on 4-nitroquinoline-1-oxide-induced tongue carcinogenesis in rats [50]. Recently Cherng et al. reported that glycyrrhizic acid can prevent UVB radiation-induced carcinogenesis in mouse model via inhibiting NF-*κ*B and cyclooxygenase-2 and activating p53 and p21 to prevent DNA damage and facilitate DNA repairing. Glycyrrhizic acid was found to induce prostate cancer cell apoptosis by triggering a caspase-independent apoptotic pathway [51, 52]. Moreover, numerous studies reported that naringin and wogonin could decrease tumor-induced vascular proliferation by suppressing the release of the VEGF from human tumor cells and inhibiting tumor proliferation and induced cell cycle arrest via p21 activation [53–57]. In addition, naringenin was also reported to could decrease tumors proliferation and growth [53, 54, 58, 59]. Several other studies also indicated that vanillin can suppress invasion, metastasis, and angiogenesis through decreasing the enzymatic activity and protein expression level of MMP-9 in cancer cell [60, 61]. The antiangiogenesis effect of vanillin was also found to be associated with PI3K inhibition in human lung cancer cells [62]. In the present study, we have demonstrated naringin, naringenin, and vanillin as the indicator components of GLY (Table 1). This existence of these molecules might partially explain why GLY could significantly affect WEHI-3 cells on cell survival, migration, and differentiation. 

We examined the antileukemia effects of GLY by using both *in vitro*, and *in vivo* assays. Our results revealed that GLY could markedly inhibit cell viability (Figure 1(a)), induce cell cycle arrest in G0/G1 phase, decrease the cell number in S phase (Figure 1(b)), and reduce cell migration (Figure 3). Besides, GLY also decreased the expression level of p-FAK, p-Akt, p-ERK, p-Rb, MMP-2, and MMP-9 whereas increased p21 expression to attenuate leukemia cell growth and migration. Moreover, we also observed that here was a significant decrease in the weight of the tumor, liver, and spleen in the GLY-treated group as compared with control group (Table 2). It was found that GLY administration significantly decreased the spleen index and liver index as compared to the untreated group (Table 3). These results suggest that GLY may improve the immunity function in WEHI-3 cancer model mice.

## 5. Conclusion

In conclusion, our investigation demonstrated that GLY has effective inhibitory activities in cell growth and migration of WEHI-3 cancer cells. To the best of our knowledge, this is the first study to explore the inhibitory mechanism of GLY in WEHI-3 cells proliferation and migration with reducing infiltration of leukemia cells into liver and spleens.

According to the previous reports, baicalein, chlorogenic acid, naringin, vanillin, and naringenin in GLY might play an important role to perform the tumor-suppressing effect: firstly, by reducing VEGF expression of tumor cells, such as baicalein [63]; secondary, the inhibitory effects of chlorogenic acid and vanillin on matrix metalloproteinases activity might be another mechanism to prevent cell migration and invasion [60, 61, 64]; thirdly, another component of GLY, naringenin, was found to inhibit cancer cell proliferation [58], making GLY a potential remedy for leukemia therapy. Moreover, WEHI-3 leukemia mice as an animal model were also evident for the antitumor activity of GLY. We found that GLY significantly decreased the average weight of the liver, spleen, and allograft tumor of in BALB/c mice injected with WEHI-3 leukemia cells (Table 2). In addition, the pathological analysis of the liver and spleen section indicated that GLY can reduce the infiltration of WEHI-3 cells into liver and spleens (Figures 6(A)-(c), 6(A)-(d), 6(B)-(c), and 6(B)-(d)). Based on these results, we proposed that ethanolic extract of GLY can be potential pharmacological reagent in preventing leukemia cancer cell proliferation and migration.

## Supplementary Material

GLY affect the protein expressions of MMP-2, MMP-9 and p21 in allograft WEHI-3 tumor model. The tumor samples were harvested and total proteins were extracted to examine the expression levels of MMP-2, MMP-9 and p21 proteins. Histograms of all values are expressed as the mean ± S.D. (*n*=3). ∗indicates *P*< 0.05, ∗∗indicates *P*<0.01 as compared with control group.

## Figures and Tables

**Table 1:** The HPLC separation conditions for identifying marked components within GLY.

Compounds	Mobile phase	Wavelength (nm)	RT (min)	Contents (mg/g of GLY)
Naringin	ACN : H_3_PO_4_ (0.11%, pH 2.2) = 22 : 78	280	10.83	13
Naringenin	ACN : H_3_PO_4_ (0.11%, pH 2.2) = 35 : 65	288	8.58	0.32
Vanillin	ACN : H_3_PO_4_ (0.0085%, pH 3.0) = 30 : 70	280	9.44	0.35

Total volume of 10 *μ*L samples was loaded into the HPLC cartridge using a flow rate of 1.0 mL/min to perform HPLC analysis. ACN: acetonitrile; RT: retention time.

**Table 2:** Antitumor effect of GLY in murine WEHI-3 leukemia model*. *

Organs	Groups			
	Normal control	Untreated group	0.75 g/kg GLY	1.5 g/kg GLY
Tumor		0.57 ± 0.27	0.22 ± 0.07^d^	0.21 ± 0.08^c^
Liver	1.45 ± 0.08	1.64 ± 0.06^a^	1.48 ± 0.08^c^	1.47 ± 0.05^d ^
Spleen	0.09 ± 0.01	0.14 ± 0.03^b^	0.14 ± 0.02	0.11 ± 0.02^c^

Data was presented as grams weight and expressed as the mean ± SD (*n* = 7). GLY extract was orally administered one time daily for 14 days. Results: ^a^ *P* < 0.05 versus normal control (without tumor graft), ^b^ *P* < 0.01 versus normal control, ^c^ *P* < 0.05 versus untreated group (tumor graft alone), and ^d^ *P* < 0.01 versus untreated group.

**Table 3:** Effect of GLY on ratio of mice organ weight/body weight in murine WEHI-3 leukemia model*. *

Organs index	Groups			
	Normal control	Untreated group	0.75 g/kg GLY	1.5 g/kg GLY
Liver	5.4 ± 0.31	7.04 ± 0.24^b^	6.10 ± 0.47^b,d^	5.86 ± 0.15^b,d^
Spleen	0.33 ± 0.02	0.58 ± 0.12^b^	0.61 ± 0.11^b^	0.44 ± 0.07^a,d^

Data was presented as ratio of mice organ weight/body weight and expressed as the mean ± SD (*n* = 7). GLY extract was orally administered one time daily for 14 days. Results: ^a^ *P* < 0.05 versus normal control (without tumor graft), ^b^ *P* < 0.01 versus normal control, ^c^ *P* < 0.05 versus untreated group (tumor graft alone), and ^d^ *P* < 0.01 versus untreated group.

**Table 4:** Relative expression level of various proteins in WEHI-3 cells treated with GLY.

Proteins	GLY (mg/mL)			
	0	0.25	0.5	1
p-FAK	1	1.12 ± 0.14	0.65 ± 0.58	0.30 ± 0.19**
p-Rb	1	0.69 ± 0.45	0.38 ± 0.09**	0.23 ± 0.09**
MMP-9	1	0.82 ± 0.34	0.34 ± 0.10**	0.24 ± 0.18**
MMP-2	1	0.87 ± 0.14	0.66 ± 0.14*	0.20 ± 0.17**
p-Akt	1	1.13 ± 0.97	0.71 ± 0.71	0.24 ± 0.25**
p-ERK1/2	1	1.07 ± 0.01	0.41 ± 0.23*	0.46 ± 0.33*
p21	1	6.00 ± 4.44	5.273 ± 2.26*	5.74 ± 2.67*

Each value represents the mean ± SD of triplication. * and **indicate *P* < 0.05 and *P* < 0.01 as compared with control group (untreated) of each group, respectively.
