# Supplementary material for: Gan-Lu-Yin Inhibits Proliferation and Migration of Murine WEHI-3 Leukemia Cells and Tumor Growth in BALB/C Allograft Tumor Model
Source: Evid Based Complement Alternat Med. 2013 Mar 17;2013:684071. doi: 10.1155/2013/684071 (PMC3613066; doi:10.1155/2013/684071)
Supplement: Supplementary file 1 — GLY affect the protein expressions of MMP-2, MMP-9 and p21 in allograft WEHI-3 tumor model. The tumor samples were harvested and total proteins were extracted to examine the expression levels of MMP-2, MMP-9 and p21 proteins. Histograms of all values are expressed as the mean ± S.D. (n=3). ∗indicates P< 0.05, ∗∗indicates P<0.01 as compared with control group. [file 684071.f1.doc]

**Supplemental data**


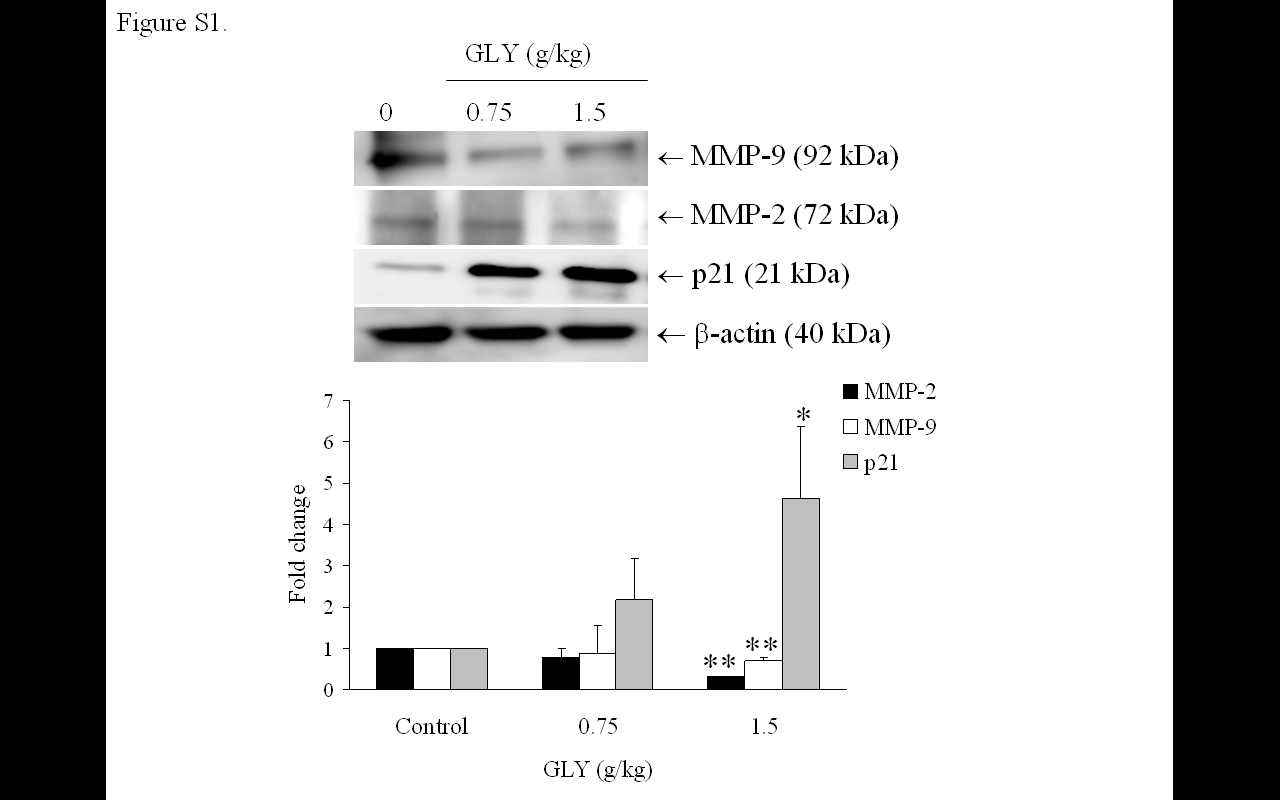


**FIGURE S1:** GLY affect the protein expressions of MMP-2, MMP-9 and p21 in allograft WEHI-3 tumor model. The tumor samples were harvested and total proteins were extracted to examine the expression levels of MMP-2, MMP-9 and p21 proteins. Histograms of all values are expressed as the mean ± S.D. (n=3). * indicate *p* < 0.05 and ** indicates *p* < 0.01 as compared with control group.
